# Turning up the adenosine turns off the spleen

**DOI:** 10.1186/1532-429X-17-S1-T1

**Published:** 2015-02-03

**Authors:** Michelle Walkden, Jennifer Bryant, Ausami Abbas, Stephen Harden, James Shambrook, Charles Peebles

**Affiliations:** 1 Cardiothoracic Radiology, University Hospital Southampton, Southampton, UK

## Background

Adenosine as a stress agent is well tolerated and has a good safety profile but occasionally when administered at 140mg/kg/min it fails to produce a haemodynamic or symptomatic response.

Studies have reported that between 4 - 18% patients do not respond to the standard dose^1, 2^.

Splenic switch-off is a novel way of assessing adequacy of hyperaemic response, to adenosine^3^.

The aim of this study was to assess the number of patients that required an increase in dose to either 175 mg/kg/min or 210 mg/kg/min over a 12-month period and the adequacy of this response using splenic switch-off.

## Methods

We retrospectively reviewed all patients who completed an adenosine stress scan in the period from July 2013 to July 2014. We identified those who had not responded to the standard adenosine dose requiring an increased dose. We recorded the physiological response to an increased dose of adenosine, both haemodynamic and/or symptomatic, and visually assessed the presence of splenic-switch off.

## Results

Over a year 955 patients had an adenosine stress scan. 74 patients (7.7%) did not respond to the standard dose and the dose was increased to either 175 mg/kg/min or 210 mg/kg/min. 82% (61) of the 74 patients that underwent an increased dose protocol had splenic switch off. See table 1.

**Table 1:** 

Splenic Switch-off	Haemodynamic/symptomatic response	No response
+ ve 61 (82.4%)	56 (75.7%)	5 (6.7%)

- ve 13 (17.6%)	10 (13.5%)	3 (4.1%)

Total 74	66 (89.2%)	8 (10.8%)

Of 11 patients who admitted to taking caffeine 10 (91%) had splenic switch off. 4 patients required the adenosine stopping early because of transient heart block, there were no other complications.

## Conclusions

Similar to previous literature only a small percentage of patients in our cohort needed an increased adenosine dose. The majority (82%) subsequently demonstrated splenic switch-off indicating an adequate hyperaemic response. Our findings would support the use of increasing adenosine infusion rates as being both safe and effective. We now routinely increase infusion rates in non-responders, including those who have taken caffeine.

## Funding

N/A.
